# Supplementary material for: Wide field-of-hearing metalens for aberration-free sound capture
Source: Nat Commun. 2024 Apr 8;15:3044. doi: 10.1038/s41467-024-47050-9 (PMC11001966; doi:10.1038/s41467-024-47050-9)
Supplement: Supplementary file 1 — Supplementary Information [file 41467_2024_47050_MOESM1_ESM.pdf]

Supplementary Information for
Wide field-of-hearing metalens for aberration-free sound capture

Dongwoo Lee[1,*] Beomseok Oh[1,*] Jeonghoon Park[1], Seong-Won Moon[1], Kilsoo Shin[1], Sea-Moon Kim[2], and Junsuk Rho[1,3,4,5†]

[1] *Department of Mechanical Engineering, Pohang University of Science and Technology (POSTECH), Pohang 37673, Republic of Korea*

[2] *Ocean and Maritime Digital Technology Research Division, Korea Research Institute of Ships & Ocean Engineering (KRISO), Daejeon 34103, Republic of Korea*

[3] *Department of Chemical Engineering, Pohang University of Science and Technology (POSTECH), Pohang 37673, Republic of Korea*

[4] *Department of Electrical Engineering, Pohang University of Science and Technology (POSTECH), Pohang 37673, Republic of Korea*

[5] *POSCO-POSTECH-RIST Convergence Research Center for Flat Optics and Metaphotonics, Pohang 37673, Republic of Korea*

The PDF file includes:

Supplementary Note 1: Phase surface map
Supplementary Note 2: Wide-angle response of WFOH metalenses by symmetry conversion
Supplementary Note 3: Physical limitations on the conventional zigzag channel
Supplementary Note 4: Theoretical derivation of the proposed meta-atom
Supplementary Note 5: Angle-invariant characteristics on phase and transmission
Supplementary Note 6: Experimental setup
Supplementary Note 7: Additional experimental results
Supplementary Note 8: Intensity distributions of the conventional metalens
Supplementary Note 9: Peak signal-to-noise ratio
Supplementary Note 10: Impact of thermoviscous loss and meta-atom period on the focusing performance
Supplementary Note 11: Field-of-hearing characterization and comparison with previous studies on acoustic metalenses
Supplementary Figure 1-11, and Supplementary Table 1, 2

* These authors contributed equally: Dongwoo Lee, Beomseok Oh
† jsrho@postech.ac.kr

Supplementary Note 1: Phase surface map

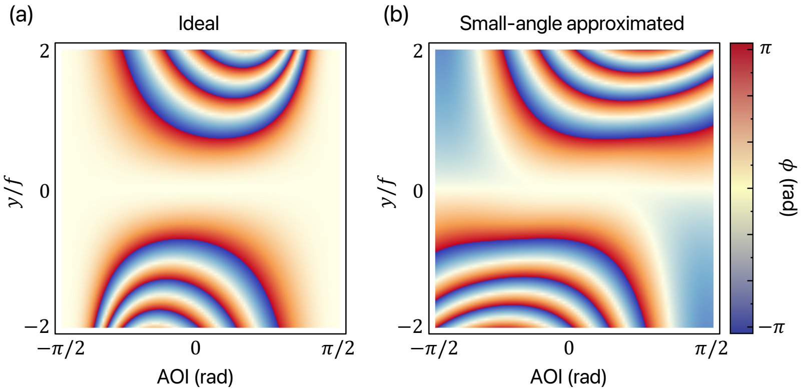

Supplementary Fig. 1: (a) ideal and (b) small-angle approximated hyperbolic phase surface maps.

We recall the angle-dependent (ideal) hyperbolic phase profile described in the main text,

$$\phi_{\text{ideal}}(r, \theta_i) = -k_0(y \sin\theta_i + \sqrt{(y - y_0(\theta_i))^2 + f^2} - \sqrt{y(\theta_i)^2 + f^2}), \tag{S1}$$

where $y_0(\theta_i)$ is equal to $f \tan\theta_i$ which is the focal-point position along the focal plane. We calculate the phase surface map with an NA of 0.9, as illustrated in Supplementary Fig. 1(a). We note that this phase profile forms an undistorted (perfect) image on the imaging plane, as it is an ideal case determined by the focus offset $f \tan\theta_i$ [S1]. However, the angle-dependent nature poses a challenge in achieving an instantaneous real-time change of phase distribution with respect to θ_i once a passive design is implemented [S1, S2]. In this context, it is highly desirable to have a phase distribution along the surface of the metalens that is independent of variations in θ_i. One way to relax the angle-dependence is by using a small-angle approximation that yields $y_0(\theta_i) = f\theta_i$ (referred to as F-theta scan lenses in optics), as calculated in Supplementary Fig. 1(b). This results in a relatively flattened phase distribution across the angle of incidence, although it comes with a distorted image mainly due to the change in $y_0(\theta_i)$. Still, in both cases, there are nonsymmetric phase distributions based on $\theta_i = 0°$, making it challenging to anticipate consistent PSF formation with varying angles of incidence. In the following note, we further provide more details about the wide-angle response of the WFOH metalens.

Supplementary Note 2: Wide-angle response of WFOH metalenses by symmetry conversion

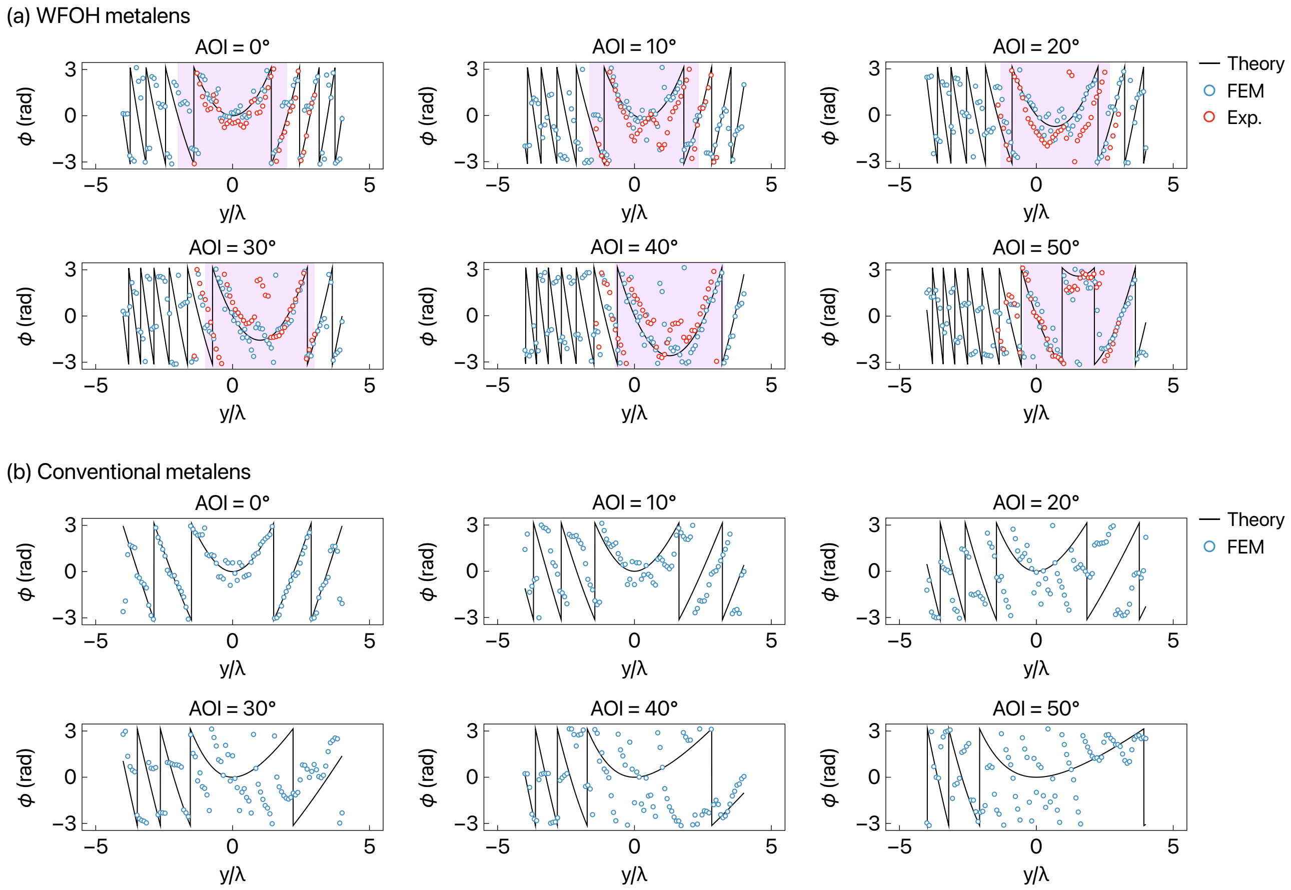

Supplementary Fig. 2: Phase distributions at the exit pupils of (a) WFOH and (b) conventional metalens with theoretical (solid lines), FEM (blue dots), and measured (red dots) results. The shaded area in (a) indicates the effective aperture (D_{eff}) and the wavevector becomes evanescent outside this region.

In this section, we delve into more details regarding the symmetry conversion based on the actual phase distributions at the exit pupils of metalenses.

As discussed in the main text, the WFOH metalens is designed with Eq. (1). The ability to achieve wide-angle focusing with angle-dispersion-free meta-atoms is attributed to the inherent characteristics of the quadratic phase. Specifically, it is owing to the effective phase shift ($\phi_{\text{PS}} = -k_0 y \sin\theta_i$) and its symmetry conversion property, which is induced by the oblique illumination. For direct investigation about this, we show the actual phase distributions at the exit pupils of metalenses, i.e., phases of the transmitted waves (Supplementary Fig. 2).

Supplementary Fig. 2(a) represents the case of the WFOH metalens, where the blue and red markers denote the results of FEM and experiment, respectively. Also, the shaded area indicates the effective aperture area, $[-f + f\sin\theta_i,\ f + f\sin\theta_i]$. We can clearly observe an effective translational shift in the phase distribution with different AOIs regardless of the passive configuration of the designed metalens (see also the angle-invariant characteristics of meta-atoms, as shown in Supplementary Fig. 5). Despite slight discrepancies from the theory in both FEM and experimental results, we can see overall similar trends. We note that various factors may contribute to deviations in the phase distribution of an actually designed metalens from its ideal counterpart [S3], e.g., non-uniform transmittance of meta-atoms and non-local effects [S4, S5] between adjacent unit cells. Supplementary Fig. 2(b) represents the phase distributions of metalenses designed based on the hyperbolic phase profile. It is apparent that beyond 10 degrees, the desired phase distributions fail to form, resulting in the inability to generate the ideal wavefront and consequently leading to aberrations that hinder wide-angle focusing.

Additionally, we characterize the symmetric properties of the quadratic phase profile with the generalized law of refraction, which can be expressed as follows:

$$n_t \sin\theta_t - n_i \sin\theta_i = \frac{\lambda}{2\pi}\frac{d\phi}{dy} = \frac{k_y}{k_0}. \tag{S2}$$

Here, n_t (n_i) indicates the refractive index with respect to transmitted (incident) wave propagation. We only consider one background medium (air) i.e., $n_t = n_i = 1$. From Eq. (S2), we see the normalized transverse wavenumber k_y/k_0 is related to the sound refraction with metasurface-induced phase discontinuity. The normalized transverse wavenumber of the quadratic phase can be expressed by

$$\frac{k_y}{k_0} = -\left(\frac{y}{f} - \sin\theta_i\right). \tag{S3}$$

The quadratic phase maintains spatial symmetry along the axis of symmetry $f\sin\theta_i$, as indicated in Eq. (S3). Based on this and the condition $|k_y| > k_0$ for an imaginary wavevector along the acoustic axis ($k_x = \sqrt{k_0^2 - k_y^2}$), we obtain the effective aperture area as follows:

$$\begin{gathered} \frac{|k_y|}{k_0} = \left|\frac{y - f\sin\theta_i}{f}\right| > 1, \\ -f + f\sin\theta_i < y < f + f\sin\theta_i. \end{gathered} \tag{S4}$$

Therefore, the effective aperture ($D_{\text{eff}} = 2f$) varies with $f\sin\theta_i$ in accordance with AOIs.

Supplementary Note 3: Physical limitations on the conventional zigzag channel

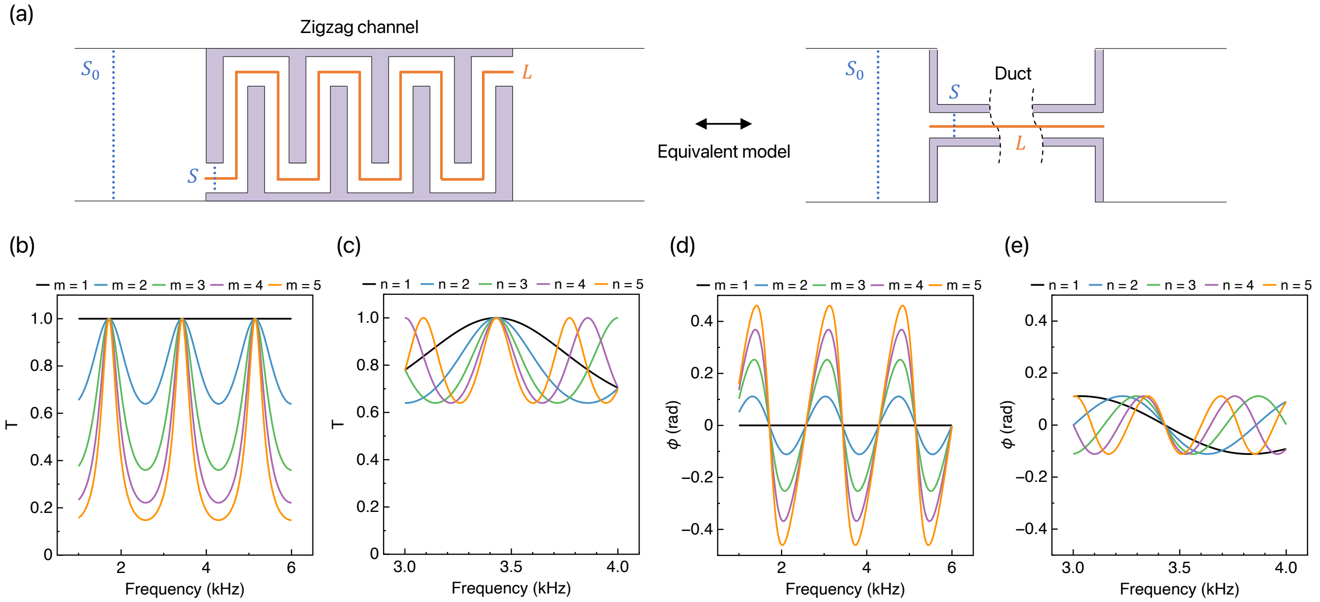

Supplementary Fig. 3: (a) Geometrically equivalent model between the zigzag channel and duct. (b),(c) T spectra modulated by m and n. (d),(e) ϕ spectra modulated by m and n.

The conventional zigzag channel design is subject to certain physical limitations that affect its performance. The underlying wave propagation characteristics of the zigzag channel can be understood within a stratified duct framework, wherein the coiling-up space is restretched, demonstrating a physical equivalence with the Fabry-Pérot resonant mechanism. Following the duct problem with the equivalent model in Supplementary Fig. 3(a), the transmission and reflection coefficients can be easily obtained as [S6]

$$\begin{aligned} t &= \frac{2e^{jkL}}{2\cos kL + j(\frac{S}{S_0} + \frac{S_0}{S})\sin kL}, \\ r &= \frac{-j(\frac{S}{S_0} - \frac{S_0}{S})\sin kL}{2\cos kL + j(\frac{S}{S_0} + \frac{S_0}{S})\sin kL}, \end{aligned} \tag{S5}$$

where S and S_0 represent the cross-sectional areas of the stretched duct and the background, respectively. L denotes the length of the duct, which was initially coiled up. Therefore, the corresponding transmission and reflectance read as

$$\begin{aligned} T &= \frac{1}{\cos^2 kL + \frac{1}{4}(\frac{S}{S_0} + \frac{S_0}{S})^2 \sin^2 kL}, \\ R &= \frac{\frac{1}{4}(\frac{S}{S_0} - \frac{S_0}{S})^2 \sin^2 kL}{\cos^2 kL + \frac{1}{4}(\frac{S}{S_0} + \frac{S_0}{S})^2 \sin^2 kL}, \end{aligned} \tag{S6}$$

where these quantities are examined to assess the efficiency and mode transition of the system. To adjust the duct geometry, dimensionless parameters are introduced: let $m = S_0/S$ and $n = L/\lambda$ as ratios. Here, λ is defined as c/f_0, where f_0 is 3.43 kHz, and c is the speed of sound at 343 m/s. For the modulation of S, we use the parameter set $(S, n) = (1, 1)$ and vary m as $m \in \{1, 2...., 5\}$ for simplicity. By using the definition $k = 2\pi f/c$, we calculate T as a function of f (Supplementary Fig. 3(b) and Supplementary Fig. 3(d)). Similarly, for the modulation of L, the parameter set is $(S, m) = (1, 1/2)$ and n varies in $\{1, 2, ..., 5\}$ (Supplementary Fig. 3(c) and (e)). Note that

$m \neq 1$ is necessary to enable nontrivial analysis, as the case of $m = 1$ corresponds to the free space scenario with $T = 1$. Modulating m leads to efficiency degradation, while modulating n causes rapid mode changes in T. The phase, calculated as $\phi = \arg(t)$, exhibits oscillations across frequencies. However, there exists a balance between T and ϕ, with ϕ tightly bounded within a small range for both m and n modulations. Therefore, the proposed unit cell in the main text, including additional Helmholtz resonators (HRs), is a good candidate to achieve arbitrary T and 2π phase modulation. It is worth mentioning that the authors, in the process of preparing their submission, have identified a horn-like metasurface that can potentially relax the aforementioned constraint, which is an interesting finding [S7].

Supplementary Note 4: Theoretical derivation of the proposed meta-atom

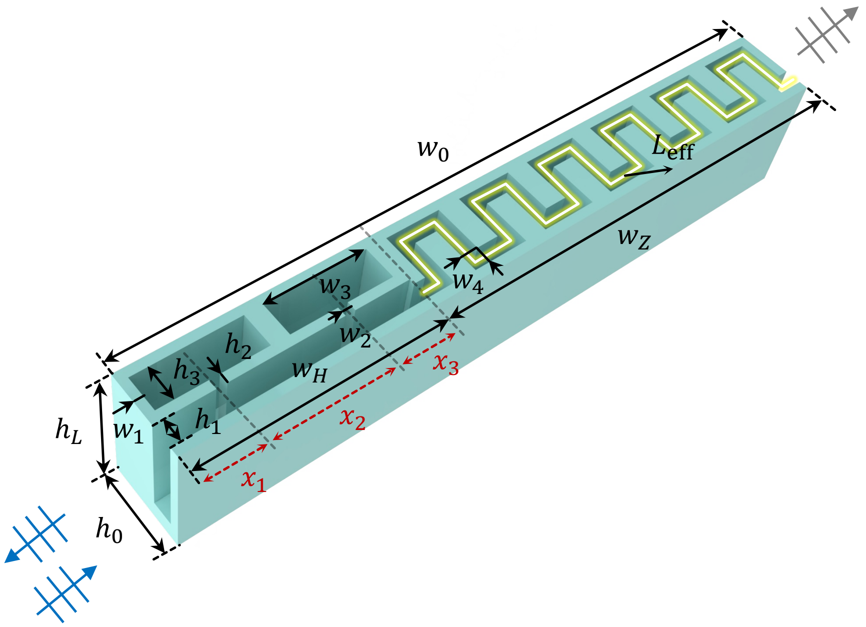

Supplementary Fig. 4: Schematic of the proposed meta-atom.

This note derives the principle and analytic model of the proposed unit cell to modulate the desired phase of the sound field based on the transfer matrix approach [S8]. Two HRs with a straight waveguide and a zigzag channel make up the two components of the proposed structure (Supplementary Fig. 4). Let the unit cell located at $0 \leq x \leq w_0$, where x_i for $i = 1, 2$ is the location of the neck of the first and second HR, for $i = 3$ is the end of the two HRs with a straight channel, and for $i = 4$ is the right end of the unit cell.

Transmission and reflection coefficients, and phase

We start by defining an incident plane wave as follows,

$$p_i = e^{-jk\cos\theta_i x - jk\sin\theta_i y}, \tag{S7}$$

where θ_i is the angle of incidence. According to Bloch's theorem, the pressure fields can be expressed in series forms,

$$\begin{aligned} p_t &= \sum_n t_n e^{-jk\beta_n y - k\alpha_n (x-w_0)} \quad \text{for } x > w_0, \\ p_r &= \sum_n r_n e^{-jk\beta_n y + k\alpha_n x} \quad \text{for } x < 0, \end{aligned} \tag{S8}$$

where t_n and r_n indicate the transmission and reflection coefficient of the nth mode of the vertical wavenumber $jk\alpha_n = k(1-\beta_n^2)^{1/2}$, and the horizontal wavenumber $k\beta_n = k\sin\theta_i + 2\pi n/h_0$, respectively. Since the existence of the transmitted and reflected waves at the inlet ($x = 0$), the particle velocity continuity condition should be satisfied,

$$u_i + u_r = u_t, \quad \text{for } 0 \leq x \leq w_0, \tag{S9}$$

where u_i, u_r, and u_t represent the incident, reflected, and transmitted waves, respectively. Substituting Eqs. (S7) and (S8) into the Euler's equation $u = j/(k\rho_0 c_0)\nabla p$ yields

$$k\alpha_n \sum_n r_n e^{-jk\beta_n y} - \alpha_0 e^{-jk\beta_n y} = \rho_0 c_0 u_t, \tag{S10}$$

where ρ_0 and c_0 are mass density and sound speed of the background medium. We can get the following relation by multiplying $e^{jk\beta_m y}$ and integrating over Eq. (S10)

$$\int_0^{h_0} \alpha_0 e^{-jk\beta_0 y} e^{jk\beta_m y} - \int_0^{h_0} r_n \alpha_n e^{-jk\beta_n y} e^{jk\beta_m y} dy = j\rho_0 c_0 \int_0^{h_1} u_t e^{jk\beta_m y} dy. \tag{S11}$$

Eq. (S11) can be simplified by exploiting the orthogonality and we get the reflection coefficient

$$r_n = \delta_{n0} - R_h U_t(0)\Phi_n(\theta_i)/k_{xn}, \tag{S12}$$

where $U_t(x) = \int u(x,y)dy$ indicates the volume velocity, $\Phi(\theta_i) = \frac{1}{h_1}\int_0^{h_1} e^{jk\beta_n y}dy$, and $R_h = \rho_0 c_0/h_0$. Similarly at the outlet ($x = w_0$), the transmission coefficient can be obtained

$$t_n = R_h U_t(w_0)\Phi_n(\theta_i)/k_{xn}. \tag{S13}$$

The acoustic pressure at the inlet can be expressed as

$$p(y,0) = 2e^{-jk\sin\theta_i y} - \sum_n r_n e^{-jy\beta_n y}. \tag{S14}$$

By adopting the averaged pressure $\bar{p_t}$, we obtain the following relation

$$\begin{aligned} 2\Phi_0^*(\theta_i) - \bar{p_t}(0) &= U_t(0)\sum_n R_h|\Phi_n(\theta_i)|^2(k_{xn})^{-1}, \\ \bar{p_t}(w_0) &= U_t(w_0)\sum_n R_h|\Phi_n(\theta_i)|^2(k_{xn})^{-1}. \end{aligned} \tag{S15}$$

Now we have the acoustic pressure and volume velocity in the region $0 \le x \le x_1$

$$\begin{aligned} p_1 &= A_1 e^{-jkx} + B_1 e^{+jkx}, \\ U_1 &= \frac{A_1 e^{-jkx} - B_1 e^{+jkx}}{R_{h_1}}, \end{aligned} \tag{S16}$$

where $R_{h_1} = \rho_0 c_0/h_1$ and $x_1 = w_2 + w_3/2$. At the inlet, the following expression can be obtained

$$\begin{pmatrix} A_1 \\ B_1 \end{pmatrix} = M_1 \begin{pmatrix} \bar{p_t}(0) \\ U_t(0) \end{pmatrix}, \tag{S17}$$

where M_1 is the transfer matrix,

$$M_1 = \begin{pmatrix} 1/2 & R_{h_1} \\ 1/2 & -R_{h_1} \end{pmatrix}. \tag{S18}$$

Likewise, the relations of the second region $x_1 \le x \le x_1 + x_2$ are as follows

$$\begin{aligned} p_2 &= A_2 e^{-jk(x-x_1)} + B_2 e^{+jk(x-x_1)}, \\ U_2 &= \frac{A_1 e^{-jk(x-x_1)} - B_2 e^{+jk(x-x_1)}}{R_{h_1}}. \end{aligned} \tag{S19}$$

The continuity condition should be satisfied at the boundary $x = x_1$,

$$\begin{aligned} p_1(x_1) &= p_2(x_1), \\ U_1(x_1) &= U_2(x_1) + U_h(x_1), \end{aligned} \tag{S20}$$

with $U_h(x_1) = p_1(x_1)/Z_h$, where Z_h is the adjusted acoustic impedance of HRs which can be derived based on the Green's function theory (see the following section "Adjusted impedance of two Helmholtz resonators array"). With Eqs. (S16) and (S20), one can express

$$\begin{pmatrix} A_2 \\ B_2 \end{pmatrix} = M_2 N_1 \begin{pmatrix} A_1 \\ B_1 \end{pmatrix}, \tag{S21}$$

$$M_2 = \frac{1}{2}\begin{pmatrix} 1-\sigma & -\sigma \\ \sigma & 1+\sigma \end{pmatrix}, \quad N_1 = \begin{pmatrix} e^{-jkx_1} & 0 \\ 0 & e^{+jkx_1} \end{pmatrix}, \tag{S22}$$

where $\sigma = R_{h_1}/Z_h$. In the same manner, the transfer matrices for other regions can be obtained as follows:

$$\begin{pmatrix} A_3 \\ B_3 \end{pmatrix} = M_2 N_2 \begin{pmatrix} A_2 \\ B_2 \end{pmatrix}, \quad \begin{pmatrix} A_4 \\ B_4 \end{pmatrix} = M_3 N_3 \begin{pmatrix} A_3 \\ B_3 \end{pmatrix}, \tag{S23}$$

$$N_2 = \begin{pmatrix} e^{-jkx_2} & 0 \\ 0 & e^{+jkx_2} \end{pmatrix}, \quad M_3 = \frac{1}{2}\begin{pmatrix} 1+\zeta & 1-\zeta \\ 1-\zeta & 1+\zeta \end{pmatrix}, \quad N_3 = \begin{pmatrix} e^{-jkx_3} & 0 \\ 0 & e^{+jkx_3} \end{pmatrix}, \tag{S24}$$

where A_3 and B_3 are related to the region between the neck of the second HR and the starting point of the zigzag part with following expressions, $R_d = \rho_0 c_0/w_4$, $\zeta = R_d/R_{h_1}$ $x_2 = w_2 + w_3$, and $x_3 = x_1$. A_4 and B_4 represent the coefficients in the zigzag channel. At the outlet i.e., $x = w_0$, the following relation can be obtained

$$\begin{pmatrix} \bar{p}_t(w_0) \\ U_t(w_0) \end{pmatrix} = M_4 N_4 \begin{pmatrix} A_4 \\ B_4 \end{pmatrix}, \tag{S25}$$

$$M_4 = \begin{pmatrix} 1 & 1 \\ 1/R_d & -1/R_d \end{pmatrix}, \quad N_4 = \begin{pmatrix} e^{-jkx_4} & 0 \\ 0 & e^{+jkx_4} \end{pmatrix}, \tag{S26}$$

where $x_4 = L_{\text{eff}} = 12(h_0 - w_1 - w_4) + \lambda/2$, and λ is working wavelength. The resulting total transfer matrix is

$$M = \overbrace{(M_4 N_4)}^{\text{zigzag}}\overbrace{(M_3 N_3)(M_2 N_2)(M_2 N_1) M_1}^{\text{HRs}}. \tag{S27}$$

From Eqs. (S15) and (S27), we finally obtain the analytic transmission and reflection coefficients as

$$\begin{aligned} t_n &= \frac{2R_h|\Phi_n(\theta_i)|^2}{\cos\theta_i}\frac{\det(M)}{m_{21}\left[\sum_n R_h|\Phi_n(\theta_i)|^2(k_{xn})^{-1}\right]^2 - (m_{11}+m_{22})\left[\sum_n R_h|\Phi_n(\theta_i)|^2(k_{xn})^{-1}\right] + m_{12}}, \\ r_n &= 1 - \frac{2R_h|\Phi_n(\theta_i)|^2}{\cos\theta_i}\frac{2\left(m_{21}\left[\sum_n R_h|\Phi_n(\theta_i)|^2(k_{xn})^{-1}\right] - m_{11}\right)}{m_{21}\left[\sum_n R_h|\Phi_n(\theta_i)|^2(k_{xn})^{-1}\right]^2 - (m_{11}+m_{22})\left[\sum_n R_h|\Phi_n(\theta_i)|^2(k_{xn})^{-1}\right] + m_{12}}, \end{aligned} \tag{S28}$$

where the transmission ($T = |t|^2$) and phase ($\phi = \arg t$) can be calculated. By adjusting h_1 and w_4, the simultaneous modulation of both T and ϕ is possible.

Adjusted impedance of two Helmholtz resonators array

As discussed, our unit cell includes two components (two HRs with a waveguide and a zigzag channel). We may consider the effects of acoustic radiation impedance in the first component (two HRs with a waveguide) because the neck of HRs radiates the acoustic energy into the waveguide [S9]. Based on Green's function, the acoustic pressure field inside the straight duct can be expressed in an integral form

$$G(x,\, y;\, x_0,\, y_0) = \sum_n \frac{\phi_n(y)\phi_n(y_0)}{2jk'_{xn}h_1} e^{-jk'_{xn}|x-x_0|}, \tag{S29}$$

where $\phi_n(y) = (2-\delta_{0n})^{1/2}\cos[k'_{yn}(y + h_1/2)]$ is the transverse eigenmode with $k'_{yn} = n\pi/h_1$ and $k'_{xn} = \sqrt{k^2 - k'_{zn}}$. By using Green's theorem, one can express

$$p(x,y) = p_i(x,y) + \int_{-w_2/2}^{w_2/2} G(x,\, y;\, x_0,\, h_1/2) \left.\frac{\partial p(x_0,y_0)}{\partial y_0}\right|_{y_0 = h_1/2} dx_0. \tag{S30}$$

Substituting the Euler's equation into Eq. (S30), we have

$$p(x,y) = p_i(x,y) - \frac{jk\rho_0 c_0}{w_2}\int_{-w_2/2}^{w_2/2} G(x,\, y;\, x_0,\, h_1/2) dx_0 U(y_0)|_{y_0=h_1/2}. \tag{S31}$$

We can get the averaged acoustic pressure at $y = h_1/2$ by using the orthogonality as follows

$$\bar{p}(y)|_{y=h_1/2} = \frac{1}{w_2}\int_{-w_2/2}^{w_2/2} p_i \, dx - \frac{jk\rho_0 c_0}{w_2^2}\int_{-w_2/2}^{w_2/2}\int_{-w_2/2}^{w_2/2} G(x,\, h_1/2;\, x_0,\, h_1/2)\, dx_0 dx\, U(y)|_{y=h_1/2}. \tag{S32}$$

From Eq. (S32), we can express the corrected radiation impedance of the neck

$$Z_d = \frac{\rho_0 c_0}{h_1 w_2^2}\left[\frac{1 - e^{-jkw_2} - jkw_2}{k^2} + \sum_n \frac{2k\left(1 - e^{-jk'_{xn}w_2} - jk'_{xn}w_2\right)}{{k'_{xn}}^3}\right]. \tag{S33}$$

Following that, we may derive the corrected impedance of the cavity. The acoustic field in the cavity ($h_2 \leq y \leq h_2 + h_3$) can be expressed in terms of the normal modes

$$p(x,y) = \sum_n \varphi_n(x)\left[A_n e^{-jk_{yn}(y-h_2)} + B_n e^{+jk_{yn}(y-h_2-h_3)}\right], \tag{S34}$$

where $\varphi_n(x) = (2-\delta_{0n})^{1/2}\cos[k_{xn}(x - w_3/2)]$. By substituting the Euler's equation into Eq. (S34), it yields

$$u(x,y) = \frac{1}{\rho_0 c_0}\sum_n \frac{k_{yn}}{k}\varphi(x)\left[A_n e^{-jk_{yn}(y-h_2)} - B_n e^{+jk_{yn}(y-h_2-h_3)}\right]. \tag{S35}$$

From the boundary condition $u = 0$ at the rigid boundary $y = h_2 + h_3$, we have

$$A_n e^{-jk_{yn}h_3} - B_n = 0. \tag{S36}$$

By using the orthogonality, the second relation between A_n and B_n can be obtained as

$$A_n - B_n e^{-jk_{yn}h_3} = \rho_0 c_0 \frac{kU(y)|_{y=h_2}}{k_{yn}w_3}\Theta_n, \tag{S37}$$

where $\Theta_n = \frac{1}{h_2}\int_{-w_2/2}^{w_2/2}\varphi_n(x)dx$. Finally, the coefficients can be expressed as

$$\begin{aligned} A_n &= \rho_0 c_0 \frac{kU(y)|_{y=h_2}}{k_{yn}w_3\left(1 - e^{-2jk_{yn}h_3}\right)}\Theta_n, \\ B_n &= A_n e^{-jk_{yn}h_3}. \end{aligned} \tag{S38}$$

Substituting Eqs. (S37) and (S38) into Eq. (S34) and averaging the resulting relation yields

$$\bar{p}(y)|_{y=h_2} = \rho_0 c_0 \sum_n \frac{kU(y)|_{y=h_2}\left(1 + e^{-2jk_{yn}h_3}\right)\Theta_n^2}{k_{yn}w_3\left(1 - e^{-2jk_{yn}h_3}\right)}. \tag{S39}$$

Finally, the corrected impedance can be written as

$$Z_c = \rho_0 c_0 \sum_n \frac{\left(1 + e^{-2jk_{yn}h_3}\right)k\Theta_n^2}{\left(1 - e^{-2jk_{yn}h_3}\right)k_{yn}w_3}. \tag{S40}$$

To obtain the whole adjusted impedance of the HRs, we may use the impedance transfer method and the resulting relation becomes

$$Z|_{y=h_1/2} = \frac{\rho_0 c_0}{w_2}\frac{Z_c + j\rho_0 c_0/w_2\tan(kh_2)}{\rho_0 c_0/w_2 + jZ_c\tan(kh_2)}. \tag{S41}$$

By adding the corrected impedance (Eq. (S40)), the whole adjusted impedance of the HRs can be obtained as

$$Z_h = \frac{\rho_0 c_0}{w_2}\frac{Z_c + j\rho_0 c_0/w_2\tan(kh_2)}{\rho_0 c_0/w_2 + jZ_c\tan(kh_2)} + j\mathrm{Im}(Z_d). \tag{S42}$$

Supplementary Note 5: Angle-invariant characteristics on phase and transmission

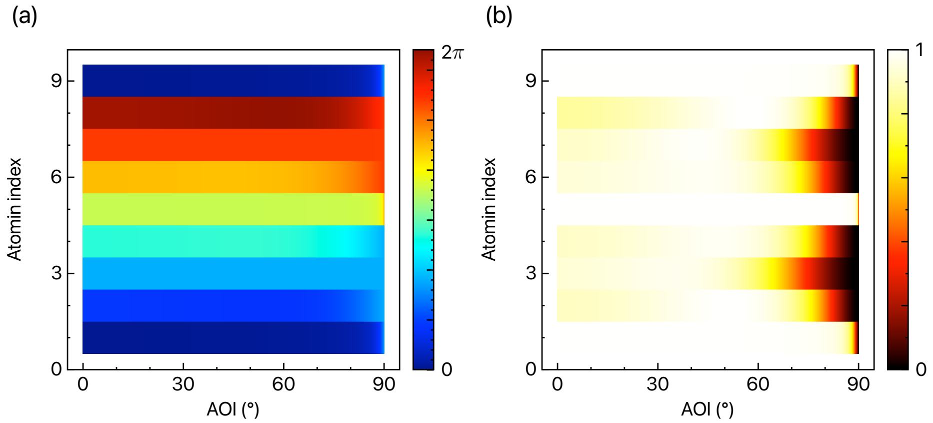

Supplementary Fig. 5: Calculations of (a) ϕ and (b) T by varying AOI in atomic indices.

Fig. 2(g) in the main text illustrates representative meta-atoms for the normal incidence. Here, we show the angle-invariant characteristics using the same nine unit cells, corresponding to 2π modulation with a step $\pi/4$. Indeed, the values of ϕ and T are not significantly dependent on the AOI. We find that the proposed meta-atoms remain valid for incident angles up to $70°$. As previously discussed in Supplementary Note 2, wide-angle focusing can be achieved without angle-dispersive properties, with the inherent nature of the quadratic phase and its symmetric conversion mechanism.

Supplementary Note 6: Experimental setup

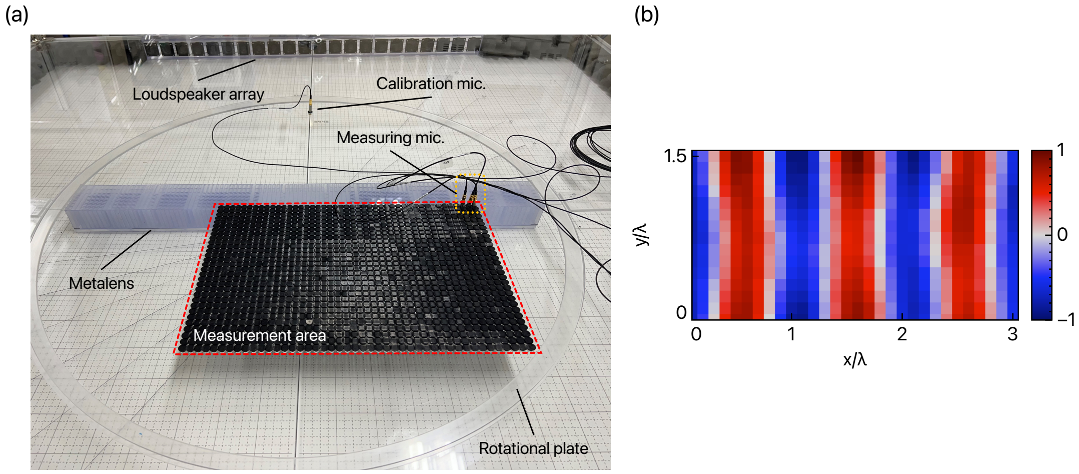

Supplementary Fig. 6: (a) Overview photograph of the experimental setup. (b) Plane wave propagation in the absence of a metalens.

As discussed in 'Results', all the measurements were conducted in a two-dimensional acoustic chamber. Supplementary Fig. 6(a) represents acoustic experimental configuration. We drilled holes at 1 cm (0.1 λ) intervals for mounting microphones on the rotational plate and carried out point-by-point measurements. The remaining openings were all sealed with rubber plugs during the measurement. Supplementary Fig. 6(b) confirms that the loudspeaker array successfully generates synchronized signals, resulting in the free space propagation of plane waves as intended.

Supplementary Note 7: Additional experimental results

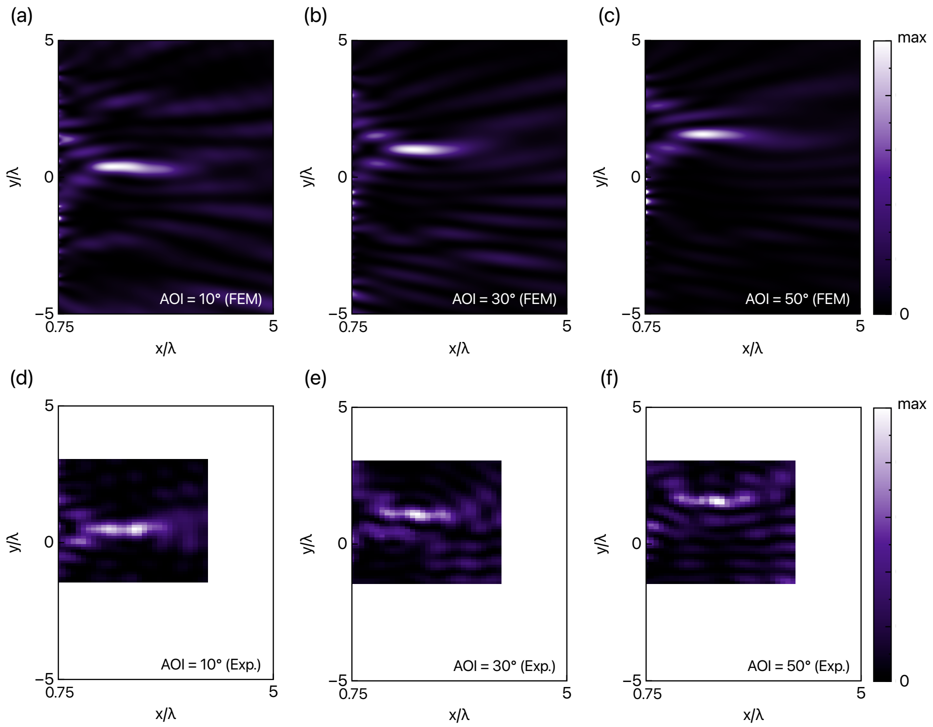

Supplementary Fig. 7: (a)–(c) Simulated and (d)–(f) experimentally measured acoustic intensity distributions at 10°, 30°, and 50° in AOIs.

We additionally include FEM and experimental results of the WFOH metalens for other AOIs (10°, 30°, and 50°) in this note due to space constraints and clarity in the main text (refer to Fig. 3 in the main text).

Supplementary Note 8: Intensity distributions of the conventional metalens

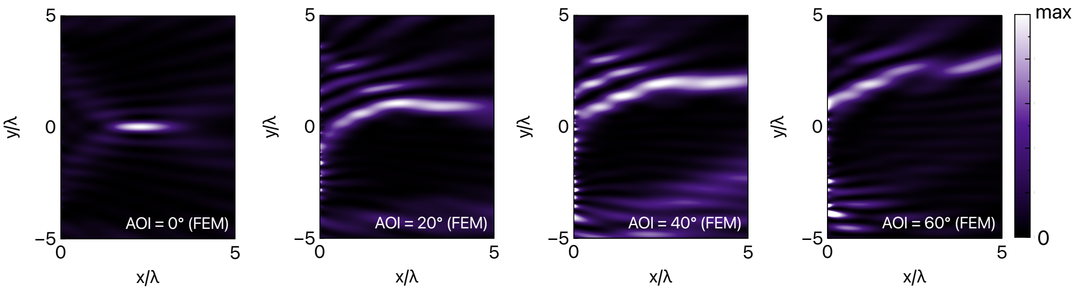

Supplementary Fig. 8: Simulated acoustic intensity distributions on conventional metalens at 0°, 20°, 40°, and 60° in AOIs.

Here, we support the intensity distribution of conventional metalens designed by the hyperbolic phase profile. It highlights the limitations of conventional metalens, showing that it only works effectively at normal incidence.

Supplementary Note 9: Peak signal-to-noise ratio

Peak signal-to-noise ratio (PSNR) stand as a widely-recognized metric for evaluating image quality [S10, S11], offering a quantitative measure of fidelity between an reference signal and a measured signal. It is calculated as a function of the mean-squared error (MSE) between the reference and measured PSFs, providing a fundamental measure for quantitative assessment of focusing quality. PSNR can be expressed as follows [S11, S12]:

$$\begin{aligned} \mathrm{PSNR} &= 20\log_{10}\left(\mathrm{Max}_i/\sqrt{\mathrm{MSE}}\right), \\ \mathrm{MSE} &= \frac{1}{n}\sum_{i=1}^{n}\left(\bar{I}_i - I_i\right)^2, \end{aligned} \tag{S43}$$

where $\bar{I}$ is the reference PSF, I is the measured PSF, and Max_i is the maximum value of the measured PSF, and n is the total number of samples. As the MSE approaches zero, indicating minimal deviation from the reference, the PSNR becomes infinity. This signifies a well-defined PSF and implies high-quality wave focusing.

To evaluate the focusing quality of our system, we calculate the PSNR for incident angles ranging from 0 to 70 degrees (see Supplementary Table 1). The calculated high PSNRs, even at large AOIs, provide clear evidence of the capability of our WFOH metalens that can be used for high-sensitivity wide-angle acoustic sensing.

Supplementary Table 1. Quantitative evaluation of PSNR

AOI (deg.)	0	10	20	30	40	50	60	70
PSNR (dB)	28.96	21.88	22.58	21.27	22.25	14.98	22.36	8.31

Supplementary Note 10: Impact of thermoviscous loss and meta-atom period on the focusing performance

Boundary-layer effects on the transmission of meta-atoms

We examine the effects of thermoviscous loss and meta-atom period on the focusing performance. Thermoviscous effects become notable when the width of the acoustic channel is reduced to less than two orders of magnitude compared to the thickness of the viscous and thermal boundary layers [S13, S14]. The effects of viscous and thermal boundary layers at the solid-fluid interface result in losses. The thickness of viscous and thermal boundary layers is defined by $\delta_v = \sqrt{2\mu/\omega\rho_0}$, $\delta_t = \delta_v/\sqrt{\mathrm{Pr}}$ where μ is the dynamic viscosity, ω is the angular frequency, ρ_0 denotes the mass density of the medium, and Pr is Prandtl number [S9, S13–S15]. At 3.43 kHz in air, δ_v is around 32 μm. In Supplementary Fig. 9(a), we present the transmission of meta-atoms considering thermoviscous effects. The average transmission in the lossless case is 0.95, and 0.79 in the presence of thermoviscous loss. Although the loss is incorporated, we find that the majority of meta-atoms exhibit $\mathrm{T} > 0.7$. Additionally, Supplementary Fig. 9(b) shows the ratio between the viscous boundary layer and the channel width ($\Gamma = \delta_v/w$). For most meta-atoms, $\Gamma < 0.02$, suggesting that w is two orders of magnitude larger than the boundary layer thickness. As a result, the impact of thermoviscous loss is not significant enough to hinder efficient wave focusing. Indeed, the calculated PSNR values are sufficiently high (refer to Supplementary Table 1), verifying that acoustic signals can be clearly resolved with high sensitivity.

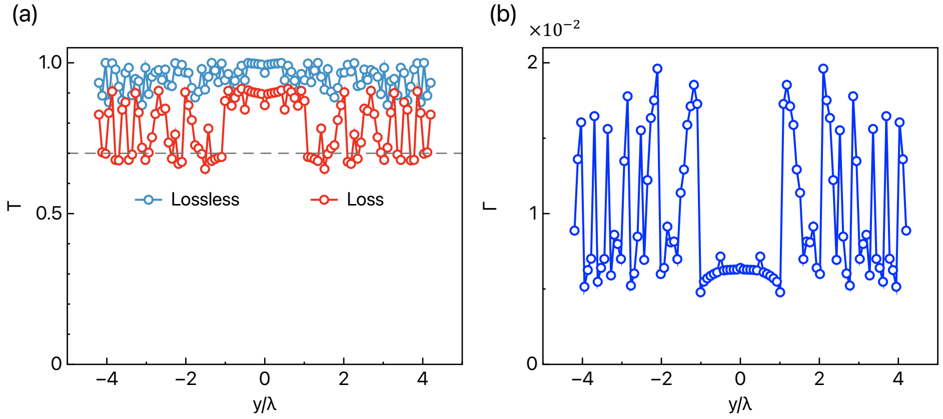

Supplementary Fig. 9: (a) Transmission of meta-atoms without loss (blue) and with thermoviscous loss (red). The horizontal dashed line indicates $\mathrm{T} = 0.7$. (b) Ratio between the viscous boundary layer and the channel width of meta-atoms, defined by $\Gamma = \delta_v/w$.

The relationship between thermoviscous effects and meta-atom period on the focusing performance

In principle, there exists a trade-off between the spatial spacing (or period, denoted as $p = h_0$ in our work) of discretized meta-atoms and thermoviscous loss. As the period p decreases, the metalens may operate closer to a *continuous* phase, potentially resulting in smaller aberrations and higher focusing efficiency. However, this proximity to continuity introduces the influence of thermoviscous effects, which can diminish efficiency due to lower transmission. Conversely, increasing p alleviates thermoviscous loss; however, larger values of p induce parasitic diffraction, consequently diminishing focusing performance.

To explore the relationship between thermoviscous effects and meta-atom period on focusing performance, we conduct numerical calculations of the PSFs relative to p, considering the thermoviscous effect. In Supplementary Fig. 10, we illustrate the normalized focusing intensity (without and with thermoviscous loss effects) and the average ratio between the viscous boundary layer and the channel width of meta-atoms (Γ_{avg}) of WFOH metalenses with periods ranging from $\lambda/9$ to $\lambda/14$. In the case without loss, we can clearly see that as p decreases, the metalens operates with a nearly-continuous phase, leading to a monotonic increase in intensity up to the theoretical limit. However, upon considering thermoviscous effects, we observe a trade-off between thermoviscous effects, which are influenced by the channel width corresponding to the meta-atom period, and discrete effects. This trade-off suggests the existence of an optimal period. Such analysis is particularly important when designing acoustic metalenses in the

ultrasonic range, where careful consideration of thermoviscous effects becomes imperative.

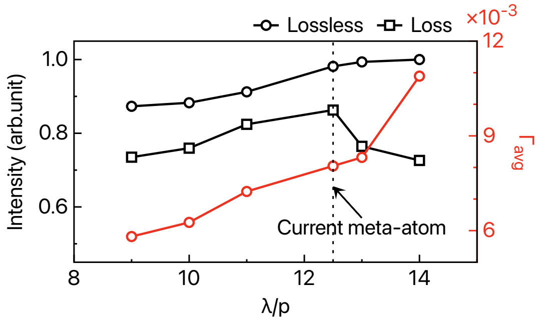

Supplementary Fig. 10: Effects of thermoviscous loss and meta-atom period on the metalens performance. Black lines with circle (square) markers represent the normalized focusing intensity without (with) thermoviscous effects, while the red line indicates the average ratio between the viscous boundary layer and the channel width of meta-atoms. For simplicitiy, the x-axis is represented as the inverse of the period.

Supplementary Note 11: Field-of-hearing characterization and comparison with previous studies on acoustic metalenses

We compare our work with previous studies on acoustic focusing using planar metasurfaces. A detailed comparison, as shown in Supplementary Table 2, highlights crucial performance metrics of the lens, particularly for the FOH capability.

Supplementary Table 2. Summary of acoustic metalenses.

Ref.	Lens type	Numerical aperture	Consideration of oblique incidence	FOH (Exp./Num.)	Meta-atom thickness (λ)	Full-width at half maximum (λ)	Focusing efficiency (%)	Operating frequency (kHz)
This work	2D, Transmissive	0.9	O	140 / 140	0.75	0.39-0.42	41-43	3.43
[S16]	2D, Transmissive	0.76	X	-	0.5	0.56	-	2.5, 4, 5.5
[S17]	2D, Transmissive	0.74	X	-	0.54	0.52	41.5	3.1
[S18]	2D, Reflective	0.51	O	- / 20	0.5	-	-	2.8-5.6
[S19]	2D, Transmissive	0.49	X	-	0.87	-	-	7.5
[S20]	3D, Transmissive	0.5	X	-	0.05	0.98	-	3.43
[S21]	2D, Transmissive	0.68	X	-	0.5	-	-	2.7, 4.1, 5.5
[S22]	2D, Transmissive	0.8, 0.94	X	-	0.65	0.52, 0.39	-	5
[S23]	3D, Reflective	0.68, 0.73	X	-	0.07	0.76, 0.74	-	3.43
[S24]	2D, Reflective	0.7	X	-	0.2	0.64-0.75	-	3, 3.5, 4.5
[S25]	2D, Reflective	0.15	X	-	0.25	0.62	-	500 (water)
[S26]	3D, Transmissive	0.87	X	-	0.98	0.55-0.83	-	8
[S27]	2D, Reflective	0.86	X	-	0.5	-	-	1.7, 3.4, 5.1
[S28]	2D, Reflective	0.78	X	-	0.21	-	-	2
[S29]	2D, Transmissive	0.79	X	-	0.87	0.59-0.7	-	1-4
[S30]	2D, Reflective	0.68	X	-	0.25	-	-	1.3, 1.9, 2.4
[S31]	3D, Transmissive	0.88	X	-	0.8	$\sim$0.6	-	5.5

To our knowledge, the determination of the size and focal length of metalenses seems arbitrary in Refs. [S16–S31],

indicating a lack of consideration for NA during the design process. This deficiency is often a result of the predominant focus on proof-of-concept demonstrations of focusing capabilities through field measurements. In contrast to the existing limitations of acoustic metalenses that encounter difficulties in achieving WFOH capabilities using the hyperbolic phase profile (commonly recognized as the trade-off between high-NA and wide FOV in conventional metalenses [S32]), our current work significantly enhances wide-angle focusing capability—an aspect often overlooked in previous studies—by emphasizing the necessity of considering the FOH. While Ref. [S18] presented results for oblique incidence, it lacked detailed interpretation with its narrow FOH.

Directly comparing the performance between previous studies and our work is laborious, as existing metalenses have often overlooked the FOH capability related to imaging evaluation metrics. Therefore, we calculate the ideal FOH as a function of the NA for both hyperbolic and quadratic phase-based metalenses. To quantitatively characterize the FOH, we introduce a function $e(\theta_i)$ that evaluates the differences between the PSFs obtained under normal and oblique incidence [S33, S34], which can be defined by

$$e\left(\theta_i\right) = \int \left| I(y,0)/\sqrt{\eta_0} - I(y-\Delta y,\theta_i)/\sqrt{\eta_i} \right|^2 dy, \tag{S44}$$

where $I(y,0)$ and $I(y-\Delta y,\theta_i)$ indicate the PSF at normal and oblique incidence, respectively. Δy denotes the spacing required to align the peak center with the reference $I(y,0)$. η indicates the focusing efficiency which is defined by $\eta = \left[\int_{-y}^{y} I|_{x=f}\, dy\right] / \left[\int_{-D/2}^{D/2} I_0|_{x=0}\, dy\right]$ integrated over the $[-y, y] = 3 \times$ FWHM region at the focal spot for each different AOIs (refer to the main text), where I_0 is the incident intensity and I is the focused intensity. We define the FOH as twice the AOI when $e(\theta_i)$ is smaller than 0.1, indicating the formation of a high-quality focusing spot.

To illustrate the calculation process of $e(\theta_i)$ schematically, we depicted an example of PSFs of the conventional metalens with an NA of 0.5 and arbitrary AOIs (Supplementary Fig. 11(a)). The left and right panels indicate the PSFs and the aligned PSFs, respectively. In Supplementary Fig. 11(b), we present the FOH performance of both conventional and WFOH metalenses, similar to findings in Refs. [S33, S34]. In particular, we observe that the FOH of the conventional metalens decreases as the NA increases, while the FOH of the WFOH metalens exhibits an extension towards the theoretical limit of 180°. In this study, we design our metalenses based on an NA of 0.9 and achieve the FOH of 140°. The achieved FOH can be mainly attributed to the limited angular responses of the proposed meta-atoms (see Supplementary Fig. 5 in Supplementary Note 5). To achieve WFOH performance closer to the ideal case, it is anticipated that considering bianisotropic and non-local effects [S4, S35] would be beneficial. However, it remains challenging to obtain such performance using conventional metalenses.

Existing metalenses [S16–S31] have not accounted for or examined oblique incidence, making direct comparisons of FOH performance with our work difficult. However, based on the analysis presented in Supplementary Fig. 11, it is evident that conventional phase-based single-layer metalenses are unlikely to achieve the WFOH capability.

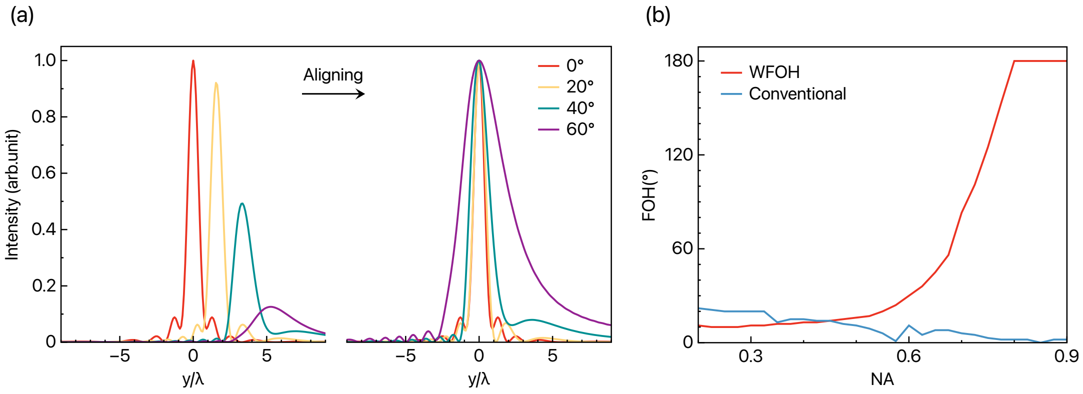

Supplementary Fig. 11: (a) Schematic illustration of the PSFs and the aligned PSFs with different AOIs. (b) Achievable FOH with respect to NA for WFOH (red) and conventional (blue) metalenses.

[S1] X. Luo, F. Zhang, M. Pu, Y. Guo, X. Li, and X. Ma, Nanophotonics **11**, 1 (2021).
[S2] A. Kalvach and Z. Szabó, JOSA B **33**, A66 (2016).
[S3] M. Zhao, M. K. Chen, Z.-P. Zhuang, Y. Zhang, A. Chen, Q. Chen, W. Liu, J. Wang, Z.-M. Chen, B. Wang, *et al.*, Light Sci. Appl. **10**, 52 (2021).
[S4] X. Wang, R. Dong, Y. Li, and Y. Jing, Rep. Prog. Phys. **86**, 116501 (2023).
[S5] K. Shastri and F. Monticone, Nat. Photonics **17**, 36 (2023).
[S6] D. T. Blackstock, *Fundamentals of physical acoustics* (Acoustical Society of America, 2001).
[S7] R. Ghaffarivardavagh, J. Nikolajczyk, R. Glynn Holt, S. Anderson, and X. Zhang, Nat. Commun. **9**, 1349 (2018).
[S8] Y. Li, S. Qi, and M. B. Assouar, New J. Phys. **18**, 043024 (2016).
[S9] P. M. Morse and K. U. Ingard, *Theoretical Acoustics*, paperback ed. (Princeton University Press, 1987).
[S10] F. Zhao, Z. Shen, D. Wang, B. Xu, X. Chen, and Y. Yang, Photonics Res. **9**, 2388 (2021).
[S11] A. Hore and D. Ziou, in *2010 20th international conference on pattern recognition* (IEEE, 2010) pp. 2366–2369.
[S12] W. Cheng, Y. Wang, Y. Zhang, H. Chen, Z. Lu, F. Zhao, Y. Wang, J. Wu, and J. Yang, Nano Lett. **24**, 254 (2023).
[S13] G. Ward, R. Lovelock, A. Murray, A. P. Hibbins, J. R. Sambles, and J. Smith, Phys. Rev. Lett. **115**, 044302 (2015).
[S14] X. Jiang, Y. Li, and L. Zhang, J. Acoust. Soc. Am. **141**, EL363 (2017).
[S15] T. Yazaki, Y. Tashiro, and T. Biwa, Proc. R. Soc. A: Math. Phys. Eng. Sci. **463**, 2855 (2007).
[S16] C. Kim, J. Kim, and W. Jeon, J. Sound Vib. **529**, 116910 (2022).
[S17] W. Li, F. Meng, and X. Huang, Appl. Phys. Lett. **117** (2020).
[S18] P. Wang, G. Yu, Y. Li, X. Wang, and N. Wang, New J. Phys. **22**, 023006 (2020).
[S19] F. Zhang, E. Perkins, S. Wang, G. T. Flowers, and R. N. Dean, Appl. Phys. Express **12**, 087002 (2019).
[S20] J. Chen, J. Xiao, D. Lisevych, A. Shakouri, and Z. Fan, Nat. Commun. **9**, 4920 (2018).
[S21] S. Tang, B. Ren, Y. Feng, J. Song, and Y. Jiang, J. Appl. Phys. **129** (2021).
[S22] K. Gong, X. Wang, H. Ouyang, and J. Mo, J. Phys. D: Appl. Phys. **52**, 385303 (2019).
[S23] S. Qi and B. Assouar, J. Appl. Phys. **123** (2018).
[S24] N.-L. Zhang, S.-D. Zhao, H.-W. Dong, Y.-S. Wang, and C. Zhang, Appl. Phys. Lett. **120** (2022).
[S25] X. Jiang, Y. Li, D. Ta, and W. Wang, Phys. Rev. B **102**, 064308 (2020).
[S26] L. Xiang, L. Jian, and H. Xinjing, IEEE Sens. J. **22**, 13989 (2022).
[S27] Y.-F. Zhu, X.-D. Fan, B. Liang, J. Yang, J. Yang, L.-l. Yin, and J.-C. Cheng, AIP Adv. **6** (2016).
[S28] Y. Zhu and B. Assouar, Phys. Rev. B **99**, 174109 (2019).
[S29] H.-W. Dong, C. Shen, S.-D. Zhao, W. Qiu, H. Zheng, C. Zhang, S. A. Cummer, Y.-S. Wang, D. Fang, and L. Cheng, Natl. Sci. Rev. **9**, nwac030 (2022).
[S30] J.-K. Weng, Y.-F. Zhu, B. Liang, J. Yang, and J.-C. Cheng, Appl. Phys. Express **13**, 094003 (2020).
[S31] S.-D. Zhao, A.-L. Chen, Y.-S. Wang, and C. Zhang, Phys. Rev. Appl. **10**, 054066 (2018).
[S32] M. Pan, Y. Fu, M. Zheng, H. Chen, Y. Zang, H. Duan, Q. Li, M. Qiu, and Y. Hu, Light Sci. Appl. **11**, 195 (2022).
[S33] Y. Hongli, C. Zhaofeng, and L. Xiaotong, Opt. Express **30**, 45413 (2022).
[S34] A. Martins, K. Li, J. Li, H. Liang, D. Conteduca, B.-H. V. Borges, T. F. Krauss, and E. R. Martins, ACS Photonics **7**, 2073 (2020).
[S35] C. Pfeiffer, C. Zhang, V. Ray, L. J. Guo, and A. Grbic, Phys. Rev. Lett. **113**, 023902 (2014).
